# Recent Advances in the Synthesis and Applications of Nitrogen-Containing Macrocycles

**DOI:** 10.3390/molecules27031004

**Published:** 2022-02-02

**Authors:** Jakub Grajewski

**Affiliations:** Faculty of Chemistry, Adam Mickiewicz University, Uniwersytetu Poznańskiego 8, 61-614 Poznań, Poland; jakub.grajewski@amu.edu.pl; Tel.: +48-61-829-1668

**Keywords:** macrocycles, polyimines, polyamides, polyureas, polyazacompounds

## Abstract

Macrocyclic nitrogen-containing compounds are versatile molecules. Supramolecular, noncovalent interactions of these macrocycles with guest molecules enables them to act as catalysts, fluorescent sensors, chiral or nonchiral selectors, or receptors of small molecules. In the solid state, they often display a propensity to form inclusion compounds. All of these properties are usually closely connected with the presence of nitrogen atoms in the macrocyclic ring. As most of the reviews published so far on macrocycles were written from the viewpoint of functional groups, synthetic methods, or the structure, search methods for literature reports in terms of the physicochemical properties of these compounds may be unobvious. In this minireview, the emphasis was put on the synthesis and applications of nitrogen-containing macrocyclic compounds, as they differ from their acyclic analogs, and at the same time are the driving force for further research.

## 1. Introduction

Supramolecular interactions between macrocycles and guest molecules have been of great interest for over 30 years, and studies on the new methods of synthesis and the structural properties of macrocycles are reflected in a large number of books and papers published. In recent times, this interest is still growing, resulting in a fairly large number of review articles. It can be noticed that at the beginning of works on macrocyclic compounds, the main focus described in the publications is on their synthesis and structure studies. The synthetic problems are related both to the preparation of macrocyclic compounds and the solubility of macrocyclic products and open-chain intermediates formed during the reactions. In recent years, the research on their properties became more and more popular, and became a driving force in publications of macrocyclic compounds. These were both optical, or in the case of optically active compounds, chiro-optical properties [1,2,3,4,5], as well as inclusion [6,7], gelation, or catalytic functions. Macrocyclic compounds also turned out to be very good selective complexing [8,9] compounds, and their metal complexes also served as a catalyst [10,11]. In many cases, all the above-mentioned properties were very different from the acyclic counterparts, which increased the interest in this field of macrocycle chemistry [12]. As in the case of acyclic compounds, their characteristic structural elements were responsible for the macrocycle activity. These include aromatic and aliphatic rings, as well as fragments with restricted or blocked conformational freedom, or incorporated into the macrocyclic skeleton of heteroatoms [13]. These hetero atoms most often include nitrogen or oxygen, but there are also macrocyclic compounds that owe their unique properties to such atoms as boron, phosphorus, sulfur, and silicon.

This minireview will be focused on the functions and synthetic strategies of the most recent papers on nitrogen-containing macrocycles. Due to scope of the review, the amino-acid-derived polyamides, cyclic peptides, nitrogen-containing cyclodextrin derivatives, and aza-crown compounds will not be discussed here.

## 2. Classes of Nitrogen-Containing Macrocycles—Synthesis

**Figure 1:**
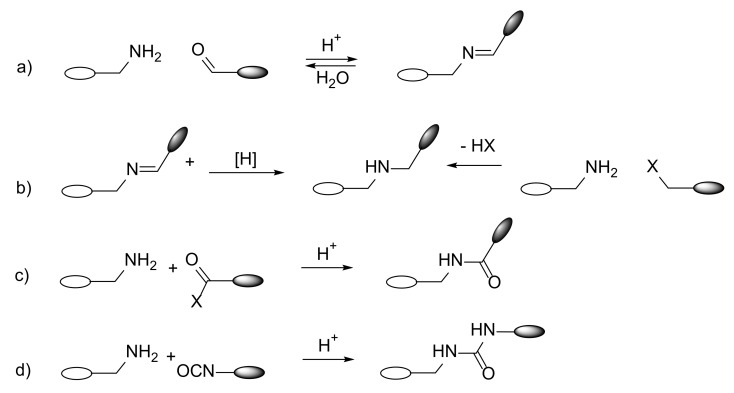
Common ring closure methods for the synthesis of nitrogen-containing macrocycles (**a**–**d**).

The type of nitrogen atom (‘aliphatic’ vs. ‘aromatic’) incorporated into macrocycles not only determines their chemical and physical properties and structure, but it also has a significant impact on the synthesis of such compounds. Depending on the bonds formed, a kinetic or thermodynamic product can be obtained. Reactions such as the formation of imines and hydrazones typically employ conditions under which the formation of products is reversible, while the synthesis of macrocyclic amides uses either high dilution or templating synthesis. During the synthesis of macrocyclic polyimines, the most frequently used reaction is the reversible formation of imine bonds with the addition of an acid catalyst. Depending on the state of thermodynamic equilibrium and the time required to reach it, water is removed from the reaction system by heating with an azeotropic water removal attachment, reacting with molecular sieves or adding inorganic hygroscopic salts or acids (Figure 1a). An analogue procedure is usually applied for hydrazone-containing macrocycles. The macrocycles containing amino groups are usually synthesized in two main ways. The first one is a reduction of the imine C=N bonds in the polyimine macrocycle, usually with NaBH_4_. Macrocyclic imine substrate can be synthesized in high yields in the process described above for imines. The second approach to nitrogen-containing macrocycles relies on direct carbon–nitrogen single-bond formation, usually performed when a secondary amine is used as a substrate. In both cases, an amine macrocycle can be later modified by substitution on nitrogen atoms, to form a tertiary amine or quaternary amine salt (Figure 1b). Usually, synthesis of amides requires diamine and diacylchloride (Figure 1c). As the amide bond formation process is nonreversible, oligomeric or polymeric byproducts can be formed. To minimize formation of acyclic products, reactions are usually carried out under high-dilution conditions to favor macrocylic products; however, yields are usually lower than in the case of macrocyclic polyimines of the similar structure. Polyurea macrocycles are built commonly by the reaction of diimines with diisocyanates (Figure 1d). Azo-group-containing macrocycles are synthetized with the azo function already incorporated in macrocyclic substrates. In this case, reactivity and photo- and thermal-induced *E*-*Z* isomerization has to be taken into account [13].

## 3. Nitrogen-Containing Macrocycles—Applications

### 3.1. Selective Ion Detection

One of the applications of macrocyclic compounds is selective anion detection. This selectivity is very important due to potential applications; e.g., in drug-production processes with metal catalysts (Pd, Ni, etc.) and recent studies on macrocycles proved that it is a promising direction [14,15]. The main goal is to obtain receptors that work under practice conditions (polar media, pH range, concentration). The more rigid structure of macrocycles than their acyclic analogues helps to control and maintain the geometry of their binding site, and makes them more selective.

**Figure 2:**
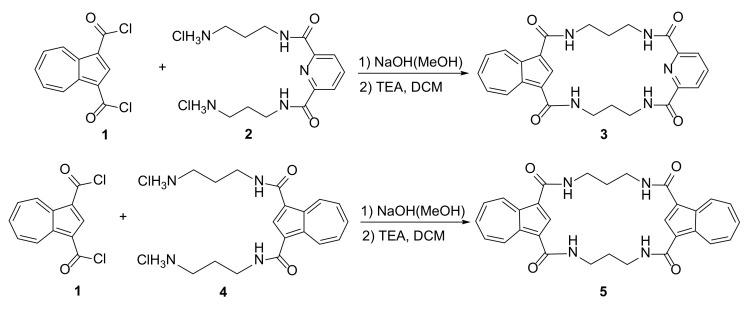
Synthesis of azulene-based receptors for phosphate anion sensing.

An interesting example of azulene-based macrocyclic receptors for recognition and sensing of phosphate anions was presented by Jurczak and coworkers [16]. The synthesis of macrocycles was performed by treating diacid dichloride **1** with appropriate diamine dihydrochlorides **2** and **4** in high-dilution conditions (Figure 2).

The conformations of **3** and **5** were studied in crystals and in solution. Their selective binding of phosphate anions (HP_2_O_7_^3−^ and H_2_PO_4_^−^) was observed, and their binding constants were calculated on the base of ^1^H NMR spectroscopy titration experiments. Compound **3** exhibits high binding constant *K*_1:1_ = 4800 for H_2_PO_4_^−^ ion in comparison to *K*_1:1_ = 270 for Cl^−^ and *K*_1:1_ = 100 for Br^−^ anions. The change of color in polar solvents such as DMSO allows the detection by eye of the interaction of macrocycle-guest molecules.

**Figure 3:**
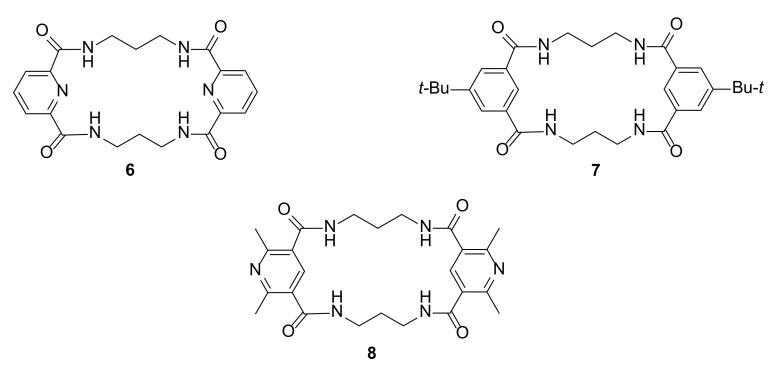
Macrocycles **6**–**8** with high affinity to chlorine binding.

Similar macrocycles designed and synthesized by Jurczak and coworkers [17] can selectively catch the chloride ion (Figure 3). The macrocycles exhibit chlorine anion binding properties that do not obey the Hofemeister series of simple anion selectivity. Investigations of shape-related properties of polyamide macrocycles **6**–**8** combined with crystal structures made it possible to identify crucial structural features that can be connected with selective chlorine binding.

The work was based on the measurement of more than 30 macrocycles and cryptands. This large library of molecules enabled the authors to observe the influence of many structural factors that affected selective anion binding properties. The size of the macrocycle and the nature of the aromatic component and additional nitrogen atoms, which provide additional hydrogen bond donors in the macrocycle structure, were taken into account. The original results were also discussed with a wide range of literature reports. These extensive studies allowed the finding of some general rules for selective Cl^−^ anion detection. The host must possess a rigid scaffold, the size of the binding pocket must not be larger than that of Cl^−^ anion incorporation, binding sites should be present from all spatial directions, and the host:guest complex must be separated from the counter ion and solvent molecules.

**Figure 4:**
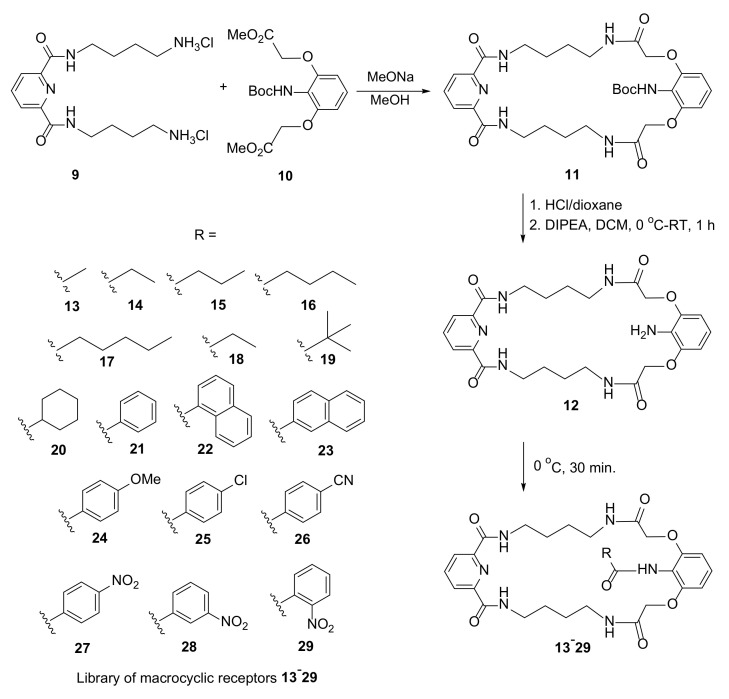
Synthesis of a library of macrocyclic polyamides **13**–**29** by static combinational chemistry.

The search for selective anion receptors via static combinational chemistry was the subject of interest of Jurczak [18]. Seventeen macrocyclic polyamides having a structure of so-called unclosed cryptands were synthesized (Figure 4). Amide functions in the macrocycle moiety formed a complex system of hydrogen-bond-donating centers.

Macrocycles **13**–**30** were also synthesized by templated (H_2_PO_4_^−^ anions in DMSO) or nontemplated concurrent syntheses with macrocyclic precursor **12** and two equimolar acid chlorides. The product and residual macrocyclic substrate ratio was tested. Stability constants for these receptors for H_2_PO_4_^−^ anions varied from 40 for **15** to 8600 for **13**.

**Figure 5:**
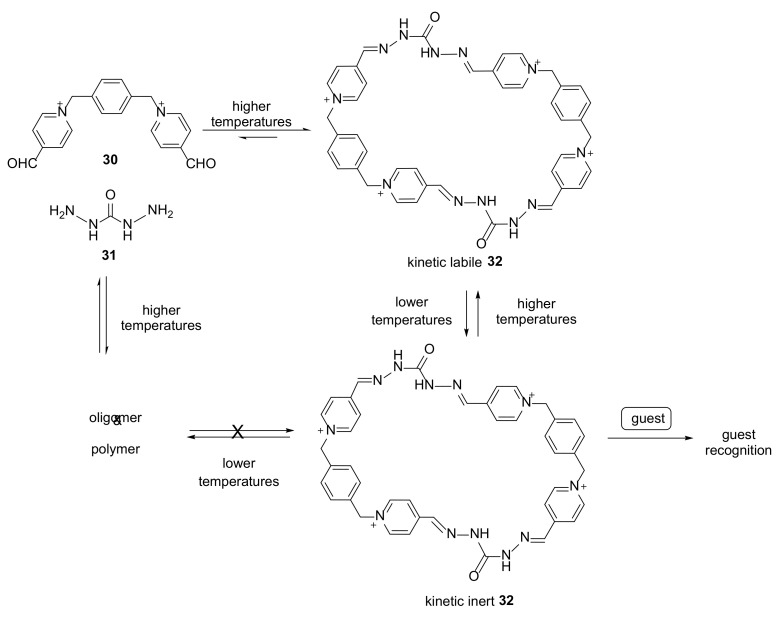
Selective and nonselective syntheses of hydrazone macrocycle **32**.

A dynamic covalent chemistry approach was used in the formation of hydrazone macrocycle self-assembly in water [19]. The reaction of **30** and **31** via reversible hydrazone condensation could produce macrocycles in high yields with interesting new electron-donor properties. A change in the reaction conditions by lowering the temperature and decreasing the acidity of the reaction mixture made macrocycle **32** kinetically inert (Figure 5).

This macrocyclic host can incorporate hydrophobic neutral or anionic guest molecules with ether groups from water solutions, and in these conditions, it is stable up to three weeks. Fine selection of the reaction conditions and their change in order to obtain a stable macrocyclic structure is a step to solving the problem of dynamic covalent chemistry; i.e., the combination of high reaction yields and stability of the final product.

Relatively small changes in the size of the macrocyclic ring containing the same functional groups may cause large changes in its conformation, and thus the structure in the crystalline phase in the properties caused by the different spatial arrangement of the donor atoms in the macrocycle ring [20].

**Figure 6:**
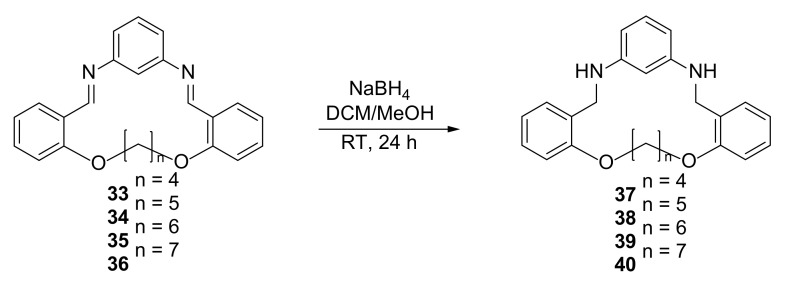
Synthesis of the series of N_2_O_2_-donor macrocycles **37**–**40**.

Reduction of macrocyclic imines **33**–**36** to the corresponding amines **37**–**40** yielded a series of N_2_O_2_ donor macrocycles. The changes in the length of flexible aliphatic chain may result in different conformation of aromatic rings, which creates the different coordination spheres. For the obtained compounds, the structures in the crystal were determined, and N-H⋅⋅⋅O interactions between neighboring molecules were found. Their affinities to mercury and iron III cations were tested, leading to conclusions that they may transport mercury (II) into the organic phase (Figure 6). 

### 3.2. Gelation Properties

Macrocycles, depending on their structure and solvent, can form crystal structures, or in some cases, gels. A recent work by Khashab and coworkers reported the self-assembly process of an enantiopure trianglimine macrocycle from gel to single crystals [21]. The understanding the mechanism of phase transitions from gel to crystal allows controlling and predicting this process. Trianglimine was synthesized in commonly used condensation reaction from optically pure 1,2-diaminocyclohexane and 4,4′-diformylbiphenyl to form a [3 + 3] condensation product. This macrocycle undergoes a guest-driven self-assembly process from gel to capsulelike or right-handed helix superstructures in solution before rearrangement to the crystal products. Helices form in the presence of toluene guest molecules, while capsulelike structures require mesitylene guests to be formed.

**Figure 7:**
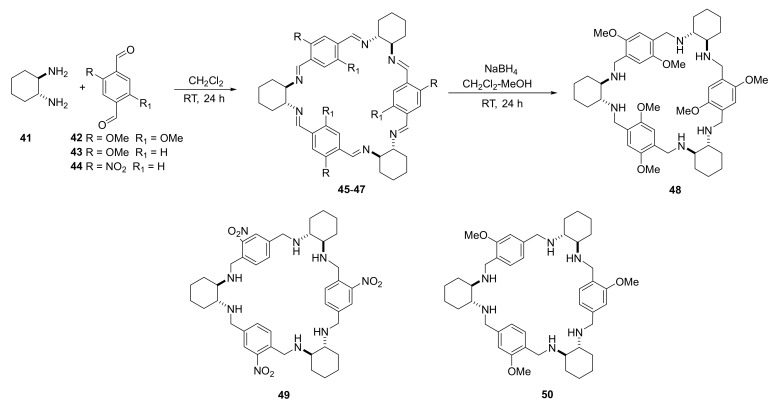
Synthesis of triangular heksaimines **46**–**47** and related heksaimines **48**–**50** displaying gelation properties.

The work of Gajewy et al. [22] presented the synthesis of triangular polyazamacrocycles—trianglimines **45**–**47** and their corresponding reduced analogues—trianglamines **48**–**50** (Figure 7). The detailed mechanism of macrocycle formation and their chiro-optical properties (ECD and VCD) were described. It was proven that the shapes of the final macrocycles were similar, but the substituents on the aromatic ring in the dialdehyde components affected the symmetry of the product.

Nitro-substituted trianglimine macrocycle **49** is an efficient low-molecular-weight supergelator. It forms stable organogels in polar media at concentrations as low as 0.4%. The reduced triangamines complexes of zinc and **48** and **50** are ligands convenient for the use in asymmetric catalysis in AHS reactions of prochiral ketones, yielding the products of enantiomeric excess up to 98%.

**Figure 8:**
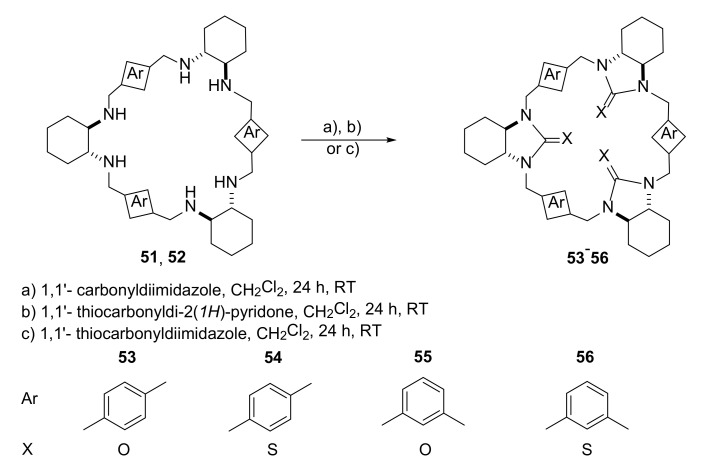
Synthesis of urea and tiourea derivatives of triaglimines **53**–**56** exhibiting gelation properties.

Gelation properties of some trianglimines [23,24] was also studied using their urea and tiourea derivatives [25]. The series of macrocycles presented in the work also differed in aromatic fragments (Figure 8). The change in urea to tiourea functions changed the conformation of the macrocycle. Detailed DFT molecular modeling studies were carried out, along with NMR measurements and X-ray crystallography.

Nonsymmetrical conformation of tiourea **56** in the solution and in the solid state was established by low-temperature NMR and by X-ray crystallography. It was found that lowering of symmetry in this molecule was crucial to the formations of metalogels with silver and copper cations. Observed gelation times varied from 1 to more than 720 min, and the gels were stable up to 30 °C.

### 3.3. Electron-Transfer Properties

**Figure 9:**
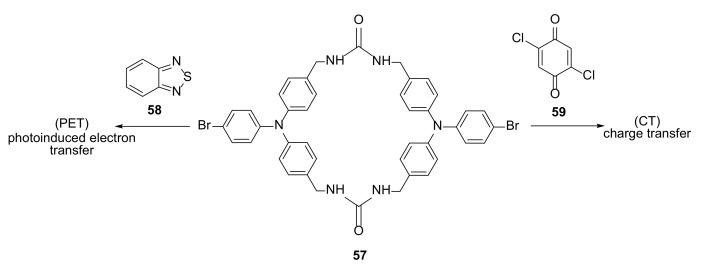
Electron-donor triphenylamine bis-urea macrocycle **57** with guest molecules enabling different electron-transfer mechanisms.

In the work of Shimizu et al. [26], the electron transfer from host to guest of the triphenyl bis-urea macrocycles were studied (Figure 9). After guest-loading into the crystal structure of **57** by the exposition to vapors of guest molecules, the electron transfer from the macrocycles to the encapsulated guest molecules **58**, **59** was observed. The observation of the crystal structures revealed how the distance and mutual orientation of host–guest molecules influenced their electron properties.

The results of diffuse reflectance spectroscopy were supported by DFT structure and absorption calculations, and allowed the detailed analysis of electronic excitations. Depending on the nature of guest molecules, the complexes displayed a charge transfer (CT) transition or photoinduced (365 nm led light) electron transfer (PET).

**Figure 10:**
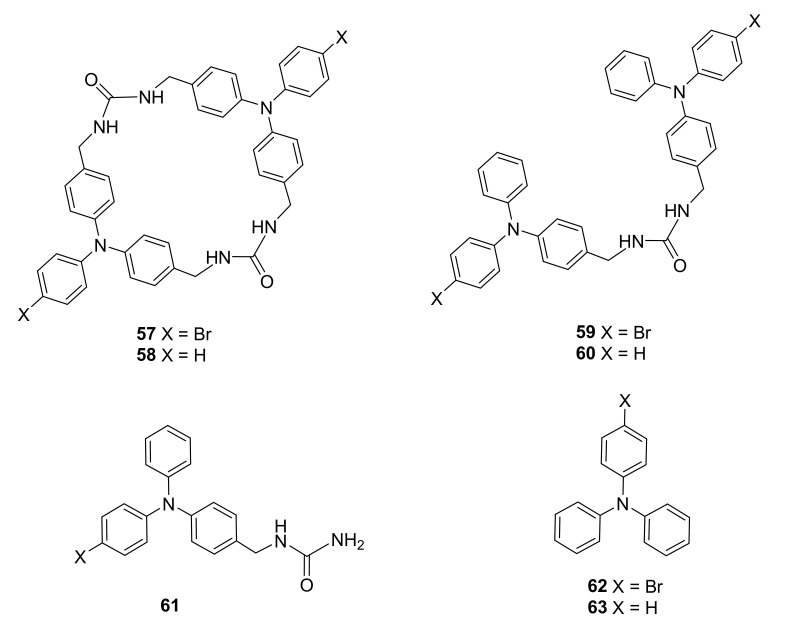
Electron-donor triphenylamine bis-urea macrocycles and their acyclic analogues containing common structural elements with selected guest molecules.

Progress of this work was presented in the recent paper [27], in which it was described how the properties of photogenerated radical cations that are characteristic to the triphenyl bis-urea macrocycles **57**–**58**, due to their porous structures in the solid phase, changed upon their self-assembly into columns in a crystal. The macrocycle void changed from 6.4 × 4.3 Å for complex of **57** with DMSO to 6.5 × 4.3 Å for complex of **58** with DME. The properties of supramolecular complexes of the triphenyl bis-urea macrocycles were also compared to their linear analogues **59**–**63** using common structural elements (Figure 10).

The EPR studies of the complexes of macrocycle **58** with guest molecules were performed to measure the approximate number of radicals generated by UV irradiation by 365 nm light. The complexes of **57** with DMF, benzene, and bromobenzene displayed the highest radical formation (up to 0.78%) after one day of irradiation.

A new class of macrocycles—molecular triangles—was presented and summarized by Stoddart and coworkers [28]. Incorporation of π-conjugated units into a macrocycle structure made them gain new optical, electronic, and magnetic properties. This class of macrocyclic compounds is composed of optically active *trans*-diaminocyclohexane (**41**) and aromatic diimides.

**Figure 11:**
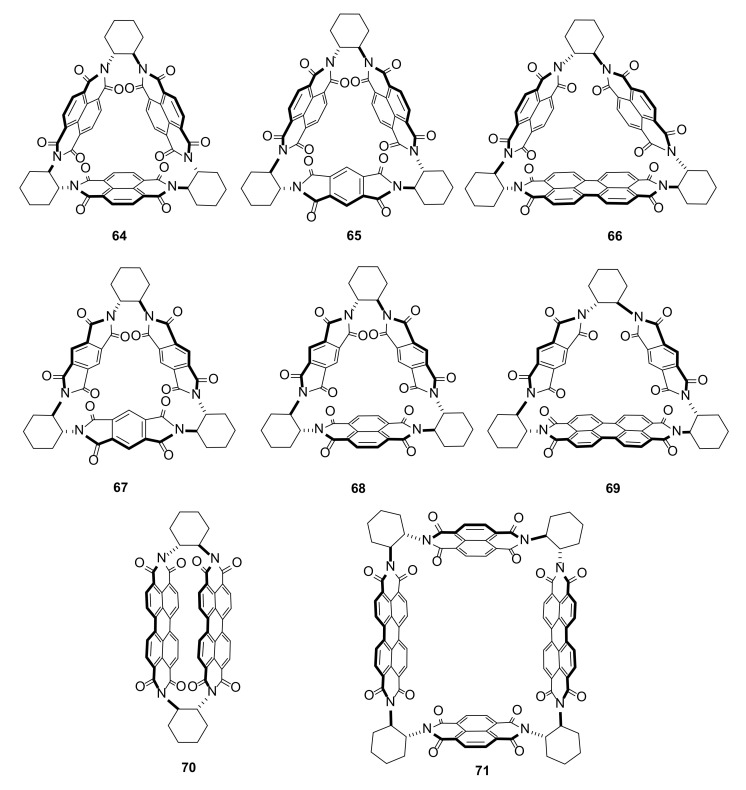
Structures of macrocyclic symmetric and nonsymmetric diimides **64**–**71** with aromatic linkers.

For the synthesis of symmetric macrocycles, one-step syntheses are presented. Construction of macrocyclic imides (Figure 11) with different aromatic units required, however, a stepwise synthesis from previously obtained diamine and aromatic dianhydride. The new compounds **64**–**71** have a set of interesting properties, such as superstructure formation with anions or electron-donor guest molecules [29]. Another property investigated by galvanostatic measurements is their potential use as an organic electrode material for large-capacity rechargeable batteries [30]. On the other hand, the solid-state luminescent properties of aromatic polyimides gained attention for their use as sensors and light emitting devices [31].

**Figure 12:**
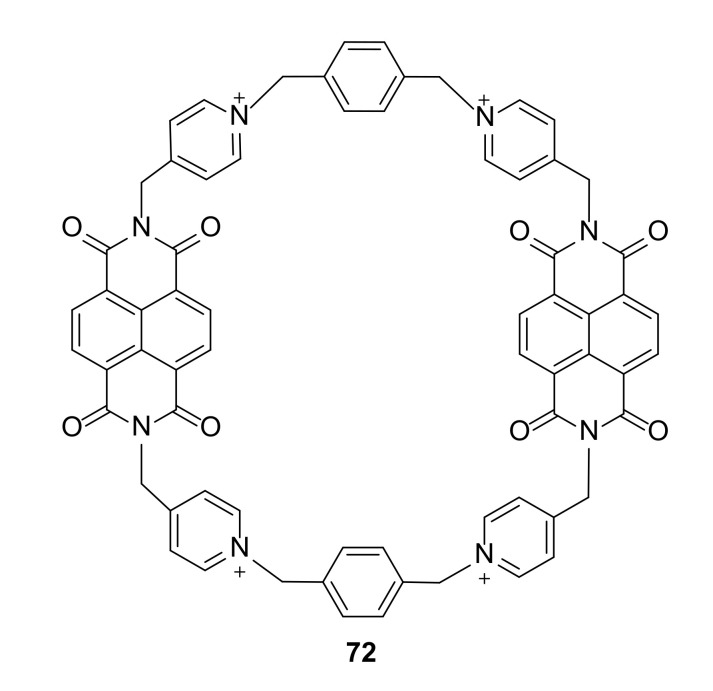
Tetrameric macrocycle **72** with bipyrridynium units displaying high conductivity.

Polyimide tetrameric macrocycles with bipyrridynium units are promising materials for organic electronics [32]. Incorporation of a naphthalene diimide moiety into a tetracationic macrocycle changes its electrochemical and electronic behavior (Figure 12).

Cyclic voltammograms show different properties of **72** in comparison to analogue acyclic compounds having the same structural components. Macrocycle **72** displayed semiconductive electron properties with high electron affinity (4.2 eV) in the solid state. A thin film of **72** displayed a maximum redox conductivity of 7.6 × 10^−4^ Sm^−1^, which makes it potentially usable in organic electronics.

Incorporation of polyaza macrocycles into macrocycle-linked covalent organic frameworks in catalyst and solvent-free conditions resulted in a successful attempt at an electrically conductive material [33].

**Figure 13:**
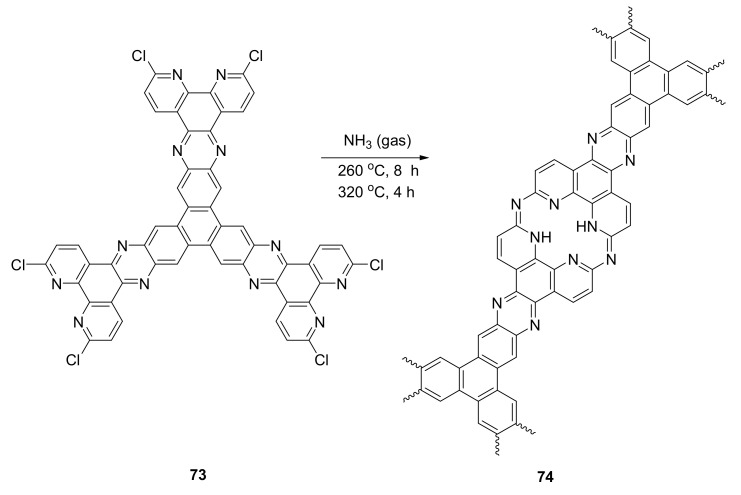
Solvent- and catalyst-free synthesis of macrocycle-linked covalent organic frameworks.

The 2-D aza-bridged bis(phenantroline) macrocycle-linked COF **74** was synthesized from *C_3_*-symmetrical tritopic monomer **73** containing three 2,9-dichloro-1,10-phenantroline units and ammonia gas, via a thermally induced reaction (Figure 13). The structure of **74** was elucidated with FT-IR, UV–vis, XPS, and ^13^C NMR analyses, and the covalent organic framework (COF) exhibited a high chemical stability toward acids and bases. After exposure to iodine vapor, COF became a semiconductive material.

The influence of nitrogen atoms in the macrocycle in comparison to an analogue structure with boron atoms instead of amino functions was presented on polycationic macrocycles, and due to their redox properties, could be used as molecular switches [34].

**Figure 14:**
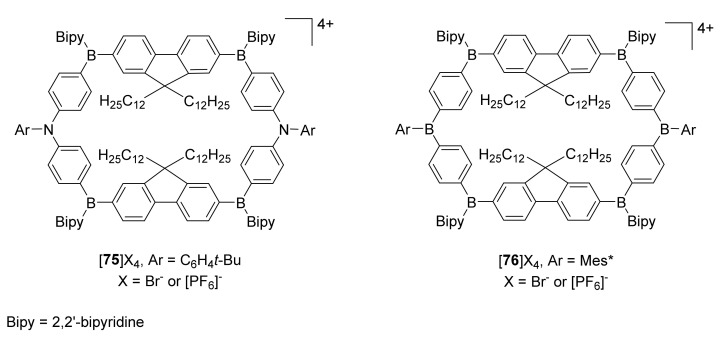
Structures of the polycationic organoboronium macrocycles **75**–**76**.

Novel polycationic macrocycles **75** and **76** possess organoboronium in their macrocyclic framework (Figure 14). The main difference between these two studied macrocycles is the presence of an electron-deficient arylborane in **76** or electron-rich arylamine moieties in **75**. Spectro-electrochemical studies showed reversible electrochromic switching between three redox states for nitrogen-containing macrocycles, or two redox states for its boronium-substituted analogue. Cyclic voltammetry results were confirmed by TD-DFT calculations that allowed assignment of electronic transitions. The switching manifested in color changes from blue to light red in **75** and blue and light yellow to dark blue in **76**.

**Figure 15:**
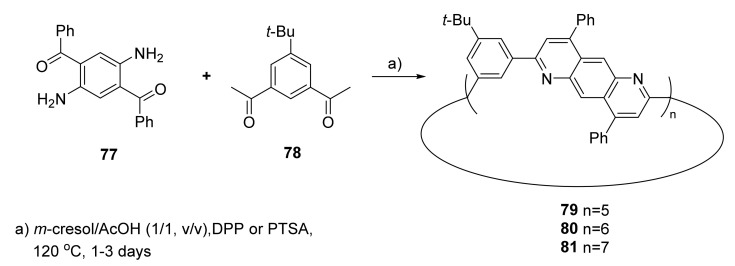
Synthetic route to 1,6-anthrazoline-containing, π-conjugated polyaza macrocycles **79**–**81**.

In some cases, electron redox or photochemical properties require the presence of large π-conjugated systems in a macrocycle structure. In this case, the nitrogen atom is a part of the aromatic ring system, as in the 1,6-anthrazoline derived macrocycle [35]. This macrocycle was synthesized by Friedlaender synthesis from **77** and **78,** which involved both imine and aldol condensation (Figure 15).

In molecules **79**–**81**, the macrocycle structures consist of all sp^2^ hybridized carbon and nitrogen atoms. UV–vis measurements of **79** and **80** in DCM solution exhibited two main absorption maxima at 403 and 407 nm. The summarized yield of all macrocycles **79**–**81** was 43%, with dominant hexameric structure **80** as the main one. The heptameric structure of **81**, due to problems with purifications caused by poor solubility, was isolated as the mixture with some oligomeric structures with similar properties.

### 3.4. Imine Bond Exchange

**Figure 16:**
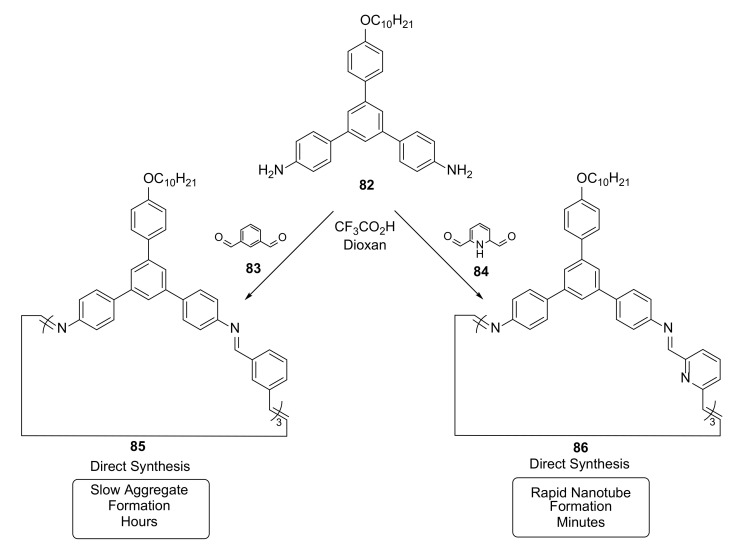
Formation and aggregation of macrocycles **85** and **86** from diamine **82** and isostructural dialdehydes **83** and **84**.

The imine-linked macrocycles also served as model compounds for studies on the mechanism of the dynamic imine bond exchange [36]. This mechanism involves not only the ratio of macrocycle to oligomer, but also includes energetic effects connected with π–π interactions of small aggregates, as well as formation of larger structures stabilized by electrostatic and solvophobic interactions (Figure 16).

Competition in the formation of macrocycles and their aggregates depends on the nature of the bisaldehyde linker. Despite the very similar structures of isophthalaldehyde **83** and 2,6-pyridienedicarboxyaldehyde **84**, their reactivities and tendencies to form nanotube structures varied by at least two orders of magnitude. The kinetically favored formation of **86** was demonstrated on the basis of a competition experiment with **85**.

### 3.5. Host–Guest Interactions with Organic Molecules

**Figure 17:**
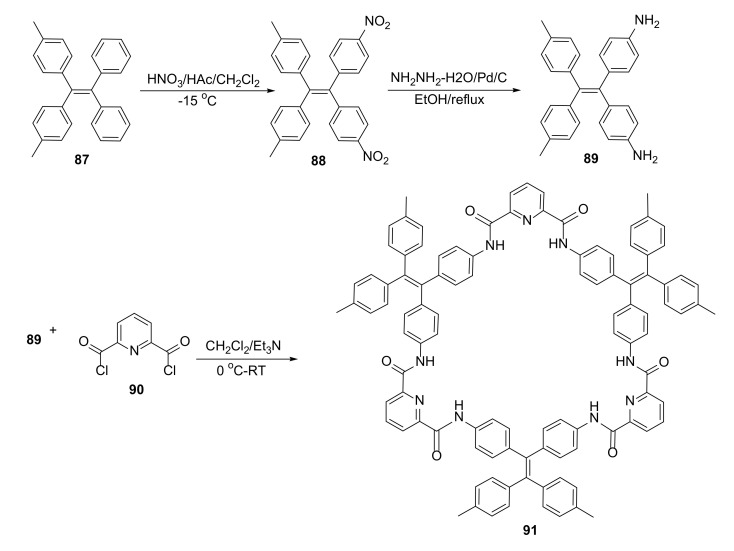
Synthesis of macrocycle **91,** which can selectively absorb TNT molecules.

The amide macrocycle, which forms a porous molecular structure and can selectively absorb guest molecules, was studied by Zheng and coworkers [37]. The synthesis of the macrocycle involved cyclization of the structurally rigid diamine **89,** containing tertaphenylethylene with dipodal acid chloride **90** (Figure 17). An X-ray analysis of the crystal structure of macrocycle **91** displayed cofacially folded tetraphenylethylene and pyridynediformyl units. Mutual insertion of two macrocycle molecules led to intertwining and catenane formation.

The porous crystal structure of the macrocycle **91** could accommodate up to two trinitrotoluene (TNT) molecules in a void, which resulted in a 1:1 guest–host ratio of compounds in the crystal. The process of TNT absorbance in the crystal pores could also be observed by fluorescence quenching. Half of the fluorescence of **91** in an H_2_O/THF suspension (C = 1.0 × 10^−7^ M) was quenched with around 10 equivalents of TNT. The pore structure of **91** was maintained, even after removal of guest and solvent molecules from the crystal.

Complexation and inclusion properties are strongly connected with the size of a macrocycle. The larger cavity can accommodate larger guest molecules; however, increasing the macrocycle size can strongly influence their solubility. This can be overcome by incorporating long alkyl chains into the macrocycle structure. An example of a polyaromatic-amine-containing macrocycle with a macrocyclic ring of a size that was capable of complexing fullerene molecules was presented by Li and coworkers [38].

**Figure 18:**
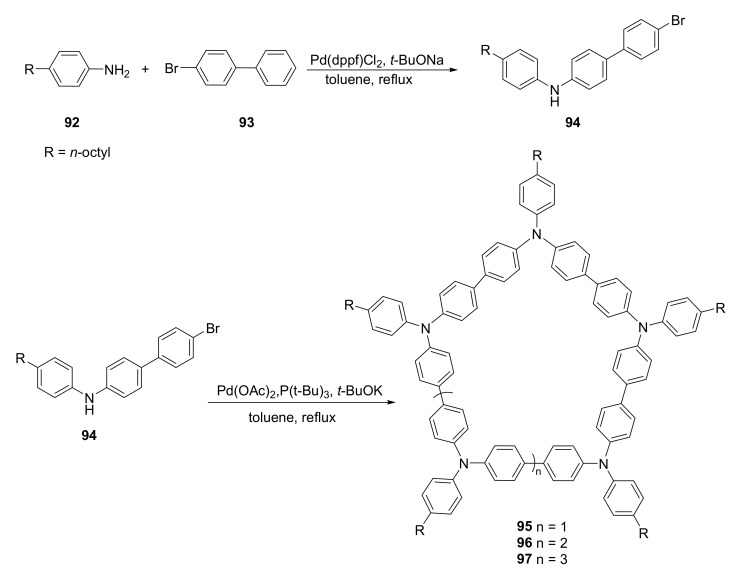
Synthesis of polyamine macrocycles **95**–**97** for selective fullerene recognition.

In one-pot synthesis, the diphenylamine derivative **94** was used as a building block to produce three macrocycles **95**–**97** with different ring sizes (Figure 18). Polymeric byproducts that formed during the reaction were removed by Soxhlet extraction. Macrocycles were then separated from their mixture by preparative gel permeation chromatography (GPC).

Host–guest interactions of the macrocycles **95**–**97** were observed by UV–vis absorption spectroscopy and fluorescence spectroscopy. Complexes of these macrocycles and C_60_, PC_61_BM, and C_70_ fullerenes were observed in 1:1 stoichiometry. The size of the macrocyclic ring influenced the C_60_, PC_61_BM complexation, while this was not observed for the C_70_ guest molecule. The complex of **95** and its complex of **95** with C_60_ fullerene were further applied in optoelectronic devices.

Molecular recognition, which is often shown in works on macrocycles as their practical application, can also be controlled. Mechanical stoppering of a guest molecule can prevent its interactions with macrocycle host-quenching molecular recognition [39]. Applying a specific impulse as a heat or addition of a base cause on cationic aromatic polyamine salts can cause controlled release of guest molecules, and enables molecular recognition. The result of this complex formation can be easily observed by the change of the color or by change of absorption in UV–vis spectra at 450 nm.

**Figure 19:**
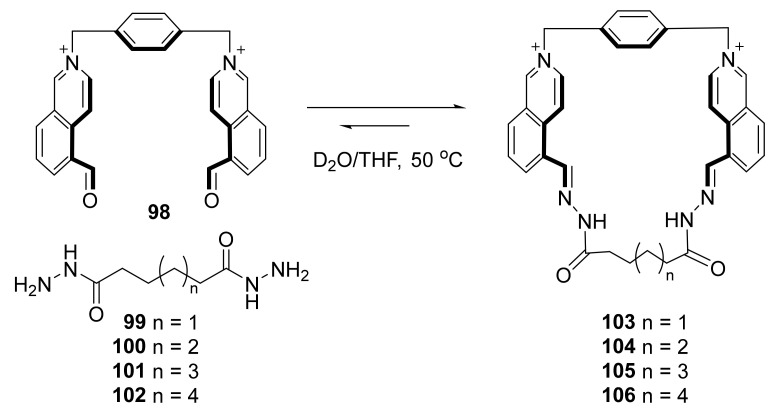
Synthesis of water-stable cationic bishydrazides **103**–**106**.

As the cyclic structure stabilizes products of hydrazide condensation in comparison to reactions performed with acyclic molecules, this reaction is also efficient when water is used as a solvent. The series of macrocycles **103**–**106** from cationic bisaldehyde component **98** and dihydrazide linkers **99**–**102** were synthesized (Figure 19) [40].

Host–guest properties of the macrocycles **103**–**106** were presented using an example of resolving a pair of isomers—phenanthrene and anthracene—which, due to their similar chemical and physical properties, were difficult to separate by commonly used methods. At higher concentrations, in more polar media and lower temperatures, the macrocycles tended to form catenanes with yields up to 33%, while at lower concentrations, mainly macrocycles were formed, with near-quantitative yields in the cases in which the template was used. It was possible to isolate compounds **103**–**106** from their mixture just by counter ion exchange, without the use of chromatography.

**Figure 20:**
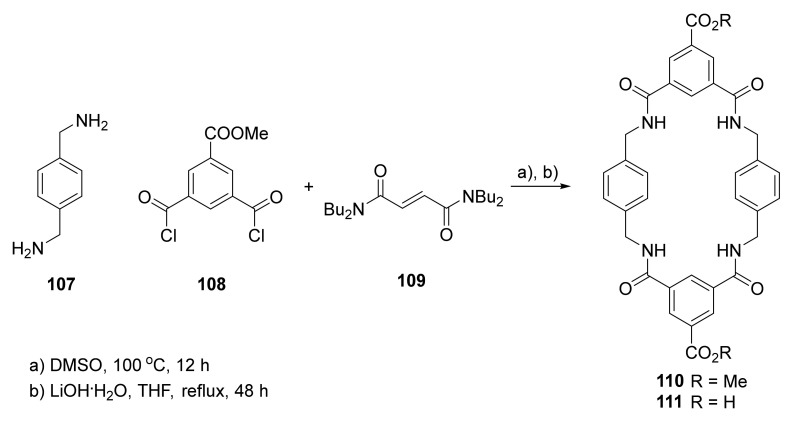
Synthesis of polyamide macrocycle **111** as a starting material for Co- or Zn-based MOFs.

Polyamide macrocyclic compounds can be used as a linkers in metal–organic frameworks [41]. The flexible benzylic amide macrocycle **111,** with two carboxylic acid groups (Figure 20), was used as an organic ligand for the synthesis of a metal–organic framework with copper (II) or zinc (II) metal ions.

Macrocycles were successfully used to form metal–organic frameworks according to commonly used solvothermal protocols, and structures of products were established by single-crystal X-ray diffraction. Both systems were evaluated as containers for the selective recognition of C_60_ and C_70_ fullerenes. Selective incorporation of the C_60_ fullerene was observed, proving these MOFs were very efficient C_60_ absorbers (34 wt %).

**Figure 21:**
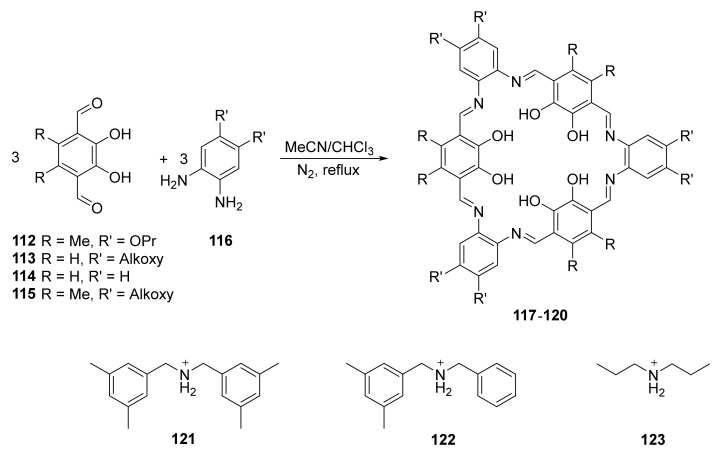
Synthesis of [3 + 3] polymimine macrocycles **117**–**120** and structures of the guest molecules’ secondary amino salts **121**–**123**.

Implementation of new functional groups into known macrocycles can change their properties and functions. The highly substituted [3 + 3] Schiff-base polyaromatic macrocycles **117**–**120** were used to form host–guest interactions with secondary ammonium-based guest molecules (Figure 21) [42].

Depending on the nature of the guest (**121**–**123**), macrocycles **117**–**120** could form internal (inside the macrocycle ring) and external complexes, or in the case of sterically crowded guest molecules such as **121,** only external ones. NMR studies of the complexes in solution were supported by theoretical calculations, and the partial tautomeric change from enol-imine partially shifted to keto-amine was observed. This tautomerization changed not only binding groups, but also conformation of the host macrocycle. Guest assembly could be also observed by bathochromic shift on UV–vis spectra from 410 to 419 nm.

**Figure 22:**
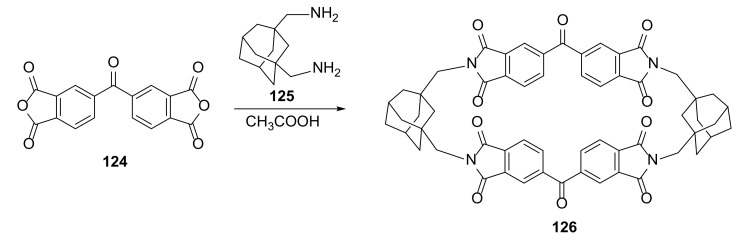
Synthesis of 2 + 2 macrocycle **126** having benzophenone-3,3′,4,4′-tetracarboxylic diimide and adamantane units.

New tetraimide macrocycle **126,** having adamantane units in its structure, was found to form inclusion crystals (Figure 22). This material, in the process of cocrystallization, could form coinclusion with the chloroform and cyclic ethers THF, 1,3-dioxane, and 1,3-dioxolane [43,44].

The structure of **126** in crystal was confirmed by X-ray crystallography. Macrocycles formed layer architectures through multiple intermolecular interactions, and guest molecules of cyclic ethers and chloroform were placed within the layer channels. The main driving forces affording formation of isostructural solvates were the van der Waals force and the CH⋯O interactions originating from the aliphatic hydrogen atoms of adamantane linkers.

**Figure 23:**
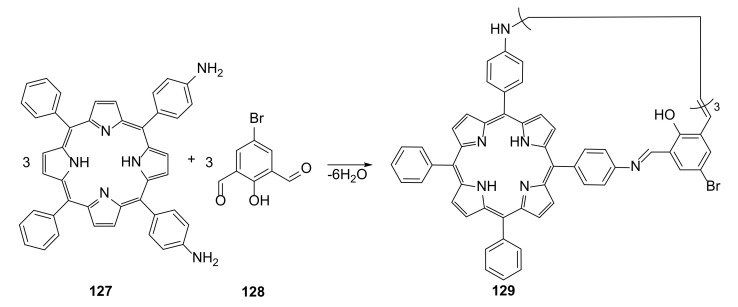
One-pot synthesis of conical phorphiryn macrocycle **129**.

An example of a giant macrocycle possessing many nitrogen atoms of different types in its structure not due to just imine bonds, but also to porphyrin moieties incorporated into its structure, was presented. A condensation reaction of *cis*-diaminophenylporphyrin with 5-bromo-2-hydroxy-1,3-benzenedicarboxyaldehyde (Figure 23) yielded [3 + 3] macrocycle **129** as the only product [45].

A detailed structural analysis was performed; due to the geometry of the substrates, the [4 + 4] or the mixture of [4 + 4] and [3 + 3] macrocycles was expected. X-ray diffraction analysis proved that the macrocycle **129** self-assembled into 1-D columns in the solid state. This structures exhibited permanent porosity, which was confirmed by several sorption experiments and powder-diffraction analysis. This was due to the presence of 1.7 nm wide 1D channels inside the column structures.

### 3.6. Ligand Exchange

**Figure 24:**
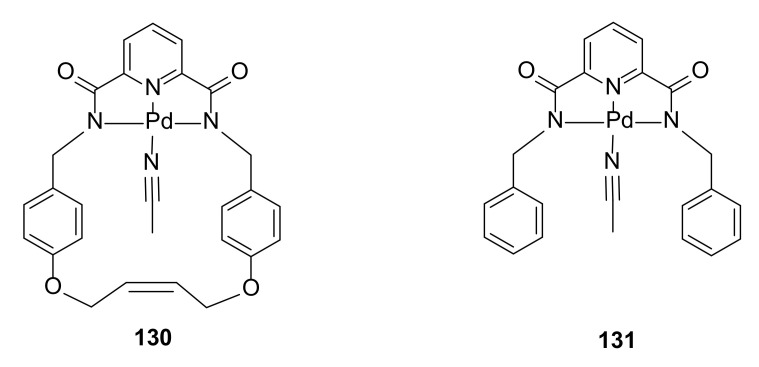
Macrocyclic palladium compound and their acyclic analogue studied for the rate of ligand exchange.

A stable macrocyclic structure can be applied for the control of ligand exchange. This phenomenon was investigated by MacLachlan and coworkers [46] using an example of a hindered palladium center built into a macrocyclic structure. Numerous ligand exchanges between acetonitrile and selected aromatic donors in macrocycle **130** were investigated by kinetic studies and compared to the results for corresponding acyclic molecule **131** (Figure 24).

Kinetic studies revealed that ligand exchange proceeded through an associative mechanism. This phenomena was connected with the small size of the macrocyclic cavity in **130**. The macrocyclic structure of the complexes slowed the rate of ligand exchange. For the bulky ligands, the rate of exchange was much slower in the macrocyclic complex **130** than in the acyclic **131**. This difference in the rate of ligand exchange faded as the size of the ligands decreased.

**Figure 25:**
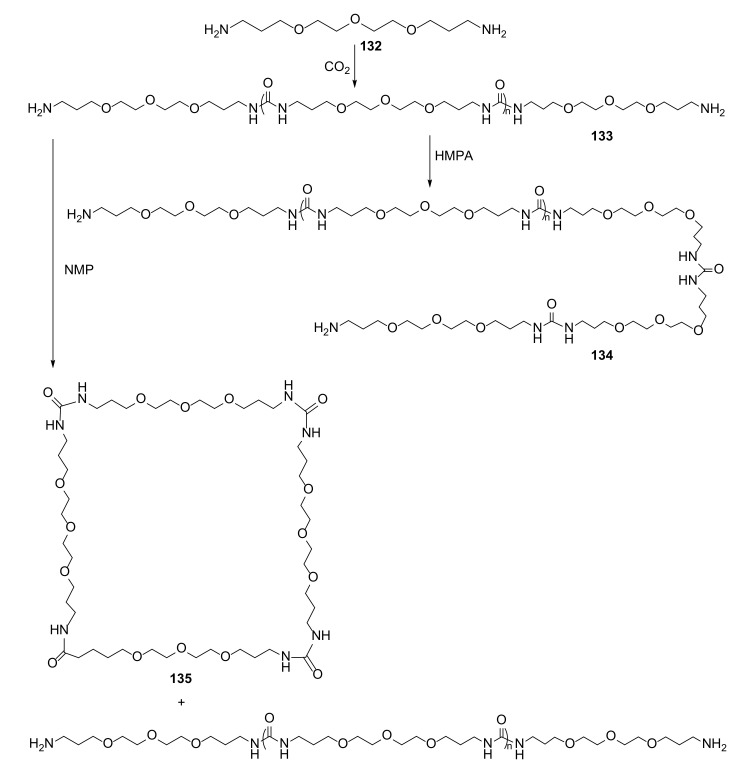
Macrocyclic polyurea **135** synthesis, according to the concept of green chemistry.

Attempts to move toward green chemistry and synthesize oligourea macrocycles from CO_2_ were also recently reported [47]. The synthesis of urea derivatives usually requires toxic isocyanates, which are produced from phosgene (Figure 25).

The polyurea oligomer **133** was prepared by reaction of diamine **132** with carbon dioxide in a pressure reactor. The cyclization (postpolymerization) o**f 133** was carried out in an atmosphere of CO_2_ with *N*-methyl-2-pyrrolidinone as an organic solvent. As carbon dioxide was used in the process, this reaction was an example of a green and sustainable approach to organic synthesis.

### 3.7. Macrocycle Size and Shape Control

**Figure 26:**
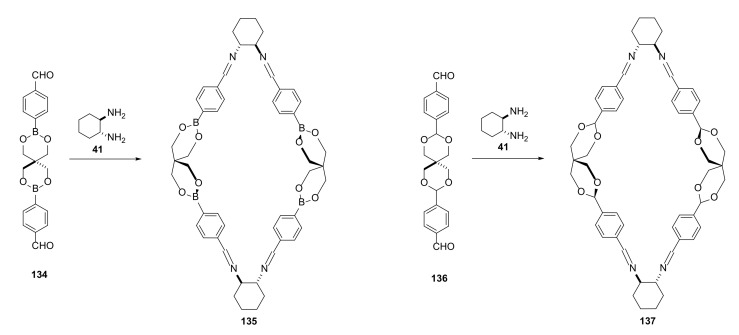
Synthesis of rhombimines with spiro boronic ester (**135**) or spiro acetal (**136**) moieties in the macrocyclic structure.

Polyimine macrocycles were also used for the introduction of axial chirality at a spiro carbon in a macrocycle ring [48]. The key step for the synthesis was the formation of an axial chirality in a quaternary carbon atom having four constitutionally identical substituents. The use of pentaerythritol for the macrocycle framework building was also a useful method for heteroatom incorporation into the macrocyclic structure (Figure 26).

Ring closure was possible by freezing the labile conformation of spiro-diboronate moiety in **134** or by diastereomeric fitting of conformationally stable spiro acetal group in **136** into a chiral macrocycle. The retention or the inversion of the absolute configuration of cyclic spiro-diboranes or separation of cyclic acetals were not observed for acyclic structures. Macrocycles **135** and **137** are very rare examples of chiral carbons having four constitutionally identical groups.

Thermodynamic equilibrium of the synthesis of enantiomerically pure polyimine macrocycles containing the spiro acetal moiety observed in the above publication showed that a mixture of racemic aldehyde components reacted with an optically pure diamine in a manner in which only one enantiomer was incorporated into the macrocycle structure. A second enantiomer of dialdehyde remained in solution, and could only form oligomeric structures.

**Figure 27:**
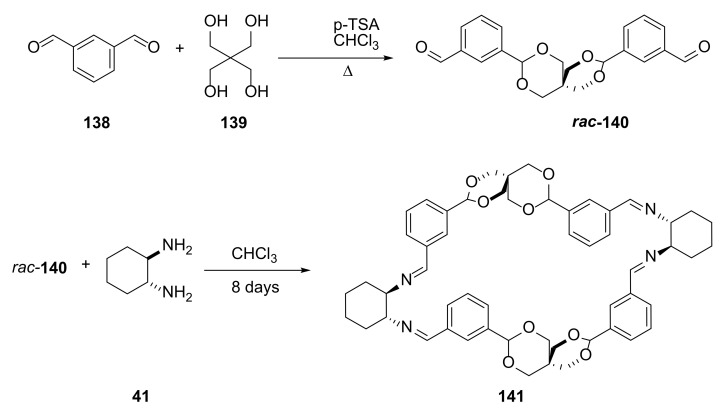
Mechanism of racemization of dialdehyde during macrocycle synthesis.

In order to invert the configuration of spiro acetal moiety, it was necessary to reversibly open at least one acetal ring to allow racemization of the dialdehyde **140** [49]. Under the reaction conditions in which water was not removed from the reaction mixture, slow hydrolysis of the acetal bond occurred, which enabled the racemization process (Figure 27).

This approach allowed the researchers to obtain the macrocyclic product **141** with an almost quantitative yield when a 1:1 ratio of dialdehyde to diamine was used. This condition of the macrocyclization, in which dialdehyde racemized, required a long time to proceed. The usual overnight heating, irrespective of the solvents used, resulted in a very complex mixture of products. Only mixing substrates **41** with **140** in a sealed test tube in which the water formed in the imine condensation process was not removed for eight days led to the formation of a product with an almost quantitative yield.

The calix[3]aramide-based cylindrical macrocycles were obtained in a poly amidation reaction of previously synthetized monomer containing two 1,3-alkylaminobenzoic acid units linked by *para*-phenylene linkers [50].

**Figure 28:**
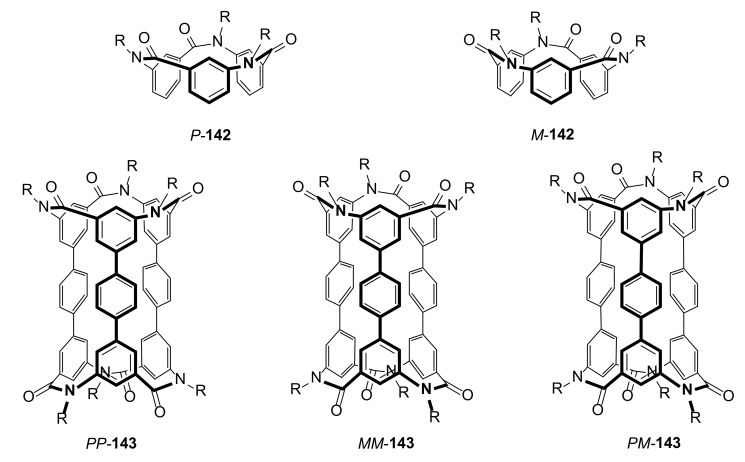
Stereoisomers of calix[3]aramide-based cylindrical macrocycle **143** caused by different mutual amide bond orientation.

The main products (**143**) were formed both in *meso* and racemic forms (Figure 28). This mixture was then separated by chiral HPLC chromatography. The ECD and VCD measurements supported by theoretical calculation helped to established configuration of pure enantiomers. Structure of macrocycle **143** was elucidated by X-ray crystallography and ^1^H NMR. The *para*-phenylene ring showed fast axial rotation at RT and down to 183 K. The absolute configurations of these compounds, as in the case of **135**, were stable due to their cyclic structures.

**Figure 29:**
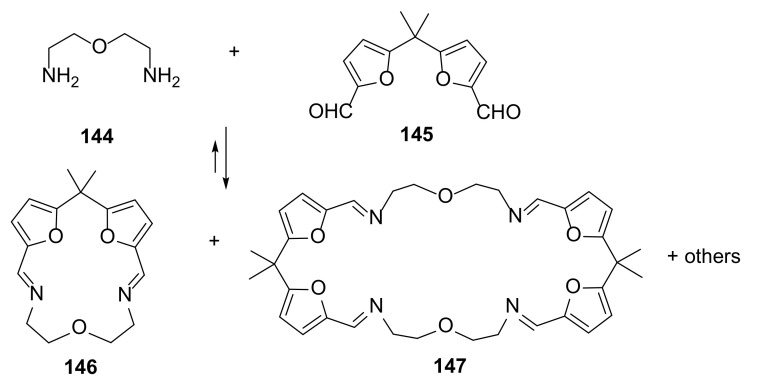
Formation of the dynamic library macrocycles **146** and **147** in acetonitrile.

A dynamic combinatorial library of macrocyclic imines allowed the observation of breaking of mirror symmetry upon spontaneous crystallization of polyimine macrocycles (Figure 29) [51].

The cascade of a reversible cyclization and crystallization process led to the formation of chiral crystals of **146**–LiCl complexes. Randomly selected crystals showed Cotton effects of the opposite sign in the solid-state electronic circular dichroism spectra (maxima at 256, 282, and 311 nm for **146**·LiCl). This was evidence that two enantiomorphic crystals were formed. The observation of this phenomenon was then broadened on the crystals formed in different temperatures and with the use of other template ions.

**Figure 30:**
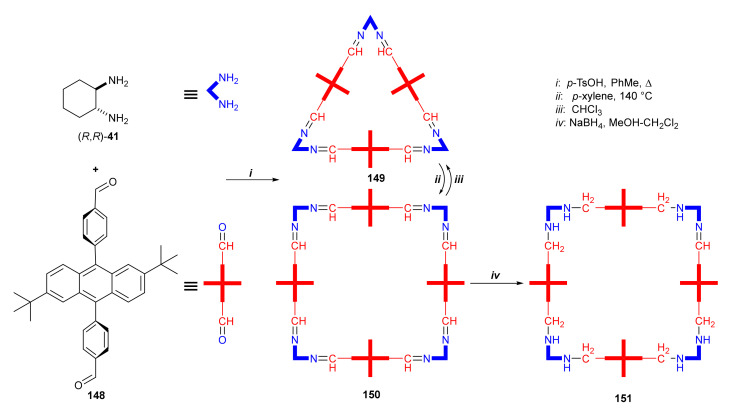
Synthesis of [3 + 3] (**149**) and [4 + 4] (**150**) macrocycles and their isomerization process.

The effect of the solvent used in the macrocyclization reaction may influence not only its time and the yield of the obtained product, but also its stoichiometry. A giant chiral square-shape octaimine macrocycle was selectively obtained from *trans*-diamnocyclohexane and 9,10-diphenylantracene-based dialdehyde (Figure 30) [52].

In most cases, reactions of substrates with such spatial orientation of the imine and aldehyde groups resulted in the formation of a macrocyclic ring in the [3 + 3] condensation reaction. Due to the steric congestion in the dialdehyde component, in this case a mixture of [3 + 3] (**149**) and [4 + 4] (**150**) condensation products was obtained. Heating in various solvents led to the discovery that only in *p*-xylene did the equilibrium of the reaction shift toward the [4 + 4] product. *p*-Xylene interacted noncovalently with the resulting macrocycle (could not be fully removed from reaction mixture). This isomerization led to an enlargement of the macrocyclic ring from a 66-membered to an 88-membered gigantocycle in which the distance between the middle points of anthracene units was 21.36 Å. Since the [4 + 4] macrocycle was re-equilibrated after dissolution to mix the [3 + 3] and [4 + 4] products, the imine bonds were reduced to obtain a chemically stable [4 + 4] octaamine **151**.

The larger polyimine macrocycles, which are usually not formed or formed in small amounts, also can be synthetized by the use of mixing the template with their smaller ring analogues. In most cases, the smallest possible ring is formed. This is related to both the usually lower solubility of the intermediates and the difficulties in the closure of the larger macrocyclic ring. The interaction of solvent molecules with the interior of the macrocyclic ring is also important.

In their studies, Lisowski, Gregoliński and colleagues showed that the phenomenon of metal complexation by the emerging macrocycle could be used to control the size of the resulting macrocyclic ring. In the condensation of *trans*-1,2 diaminocyclohexane with 2,6-diformylpyridine, a mixture of products [2 + 2] and [4 + 4] was formed [53].

**Figure 31:**
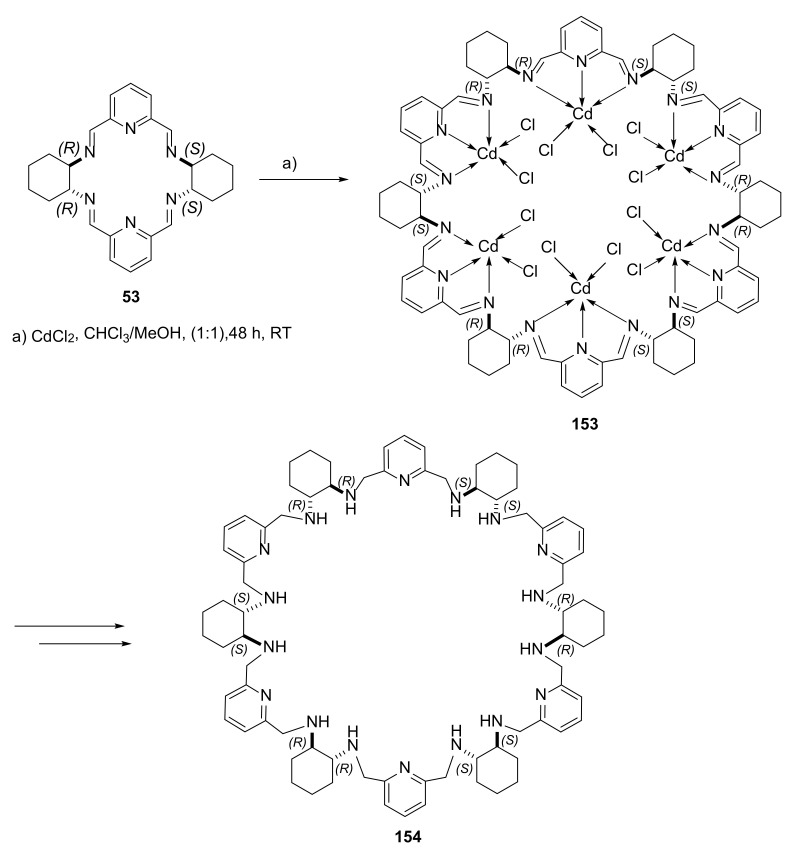
Cadmium-templated polyimine macrocycle ring expansion.

Reaction of the isolated [2 + 2] macrocycle **152** with cadmium (II) chloride resulted in the fusion of three [2 + 2] polyimines into a large [6+6] macrocycle **153** (Figure 31). In further stages of the synthesis, the obtained cadmium complex with the [6 + 6] macrocycle was reduced to the corresponding polyamine, and the coordinated cadmium was removed from the molecule. The resulting amine **154** was multiply folded, and had the ability to bind other ions or to serve as a host for small organic molecules.

**Figure 32:**
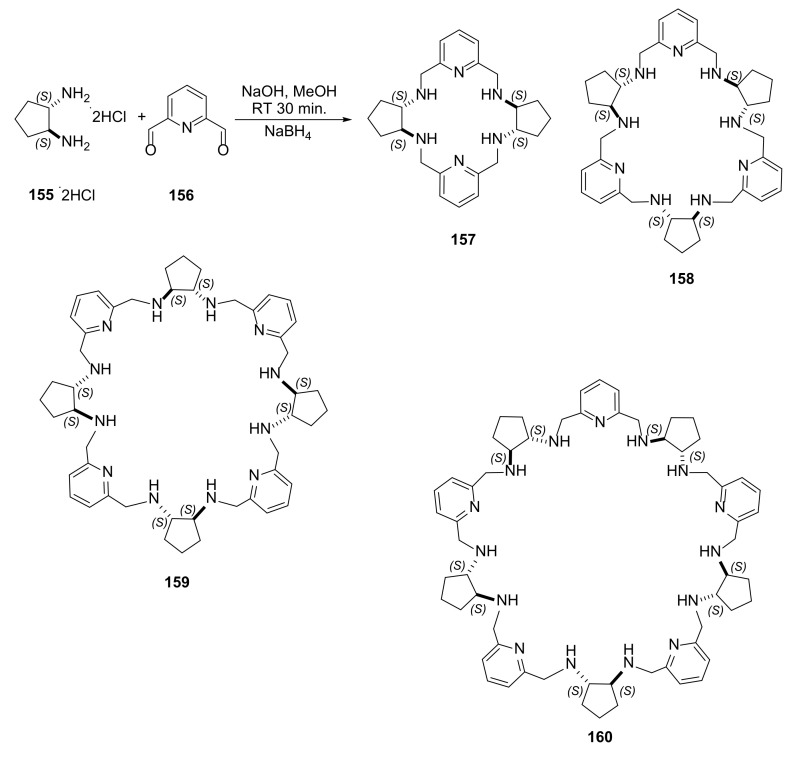
Nontemplated synthesis of the mixture of [2 + 2], [3 + 3], [4 + 4], and [5 + 5] polymimine macrocycle mixtures followed by their reduction.

The other situation can be observed in the condensation of enantiomerically pure *trans*-1,2-diaminocyclopentane (**155**) with 2,6-diformylpyridine (**156**) in methanol [54]. Reduction of the mixture of imine products results in the mixture of [2 + 2], [3 + 3], [4 + 4] and [5 + 5] macrocyclic amines **157**–**160**, which can be separated to yield small amount of new [4 + 4] and [5 + 5] products (Figure 32). The replacement of diamino component of macrocyclization from *trans*-1,2-diaminocyclopentane with the [2 + 1] primary diamine results in new a dynamic library in the condensation with 2,6-diformylpyridine with [4 + 4] and [6 + 6] cyclocondensation products.

The authors investigated the influence of chirality of the amine component on the stoichiometry of the product. The [2 + 2], [3 + 3], and [4 + 4] condensation products were formed both with racemic and enantiomerically pure diaminocyclopentane. The larger macrocycles could be obtained with cadmium (II) template reactions. Heterochiral gigantocycles, which resulted from [6 + 6] and [8 + 8] condensation, could be obtained by ring expansion reaction of a small *meso* 2 + 2 product templated by cadmium salts.

**Figure 33:**
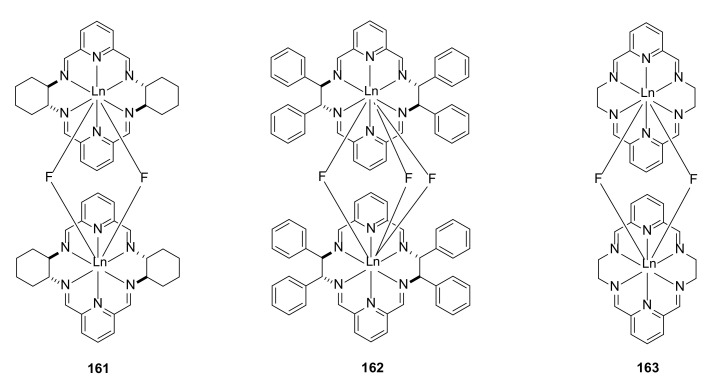
Dinuclear lanthanide (III) complexes of macrocyclic tetraimines **161**–**163**.

The sorting phenomena and the transfer of chirality was studied using the example of polyamine macrocyclic fluoride–bridged complexes [55]. The reaction of hexaaza macrocyclic complexes with lanthanide (III) and yttrium (III) ions with fluoride anions resulted in the formation of dinuclear fluoride–bridge complexes (Figure 33).

The structure indicated by X-ray single-crystal analysis showed that two metal ions bounded by macrocycles were linked by two (**161** and **163**) or three (**162**) fluoride ions, depending on the type of the macrocycle of the same size. The differences in macrocyclic complexes’ dimmer formations were explained by the more open axial coordination spheres of the complexes of hexaaza macrocycles compared to cyclen-based tetraaza macrocycles. In the case of the dimmer complexes with two different macrocyclic ligands, chirality transfer was observed by CD spectroscopy, from a chiral macrocycle complex to a nonchiral one. The structures containing trinuclear and hexanuclear lanthanide (III) complexes of chiral nonracemic macrocycles were also a subject of a study by Powell, Lisowski and coworkers [56]. The hexaaza chiral macrocycle containing three phenol groups formed trinuclear complexes, which were characterized by X-ray structure. The magnetic studies of these complexes indicated the weak ferromagnetic interactions between neighboring metal ions.

**Figure 34:**
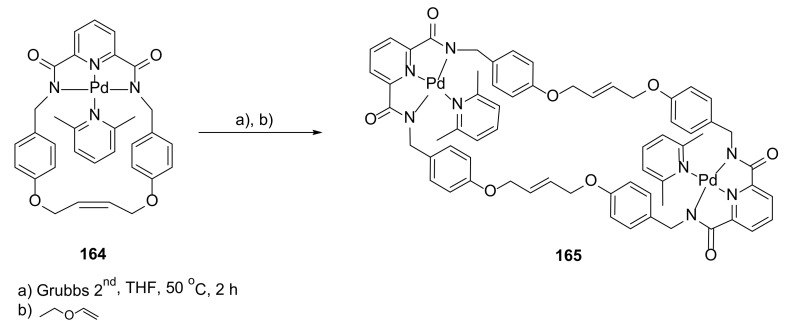
Ligand-driven ring expansion by metathesis reaction.

The ring expansion phenomenon of the unstrained palladium-containing macrocycle was a subject of a study by the MacLachan group [57]. The different strategies of ring expansion usually employ reversible reactions to form larger macrocycle with the aid of template, which change the thermodynamic distribution of macrocycles of different ring sizes. In this paper, the ring-expansion metathesis polymerization was used (Figure 34).

In this case, the driving force of the reaction was implementation of a bulky ligand into the coordination sphere of **164**. This allowed the monometallic macrocycle **164** to be transformed into the bimetallic dimeric **165** with the second generation of Grubbs catalyst, with yields up to 20%. The structure of macrocycle **165** was confirmed by ^1^H DOSY NMR and SCXRD data. The expansion of macrocycle ring was not observed with smaller ligands such as acetonitrile.

Understanding the process of crystallization and layer formation was demonstrated using an example of a tessellation process of a rigid naphthalene diimide triangle [58]. In the process of arranging triangle-shaped molecules in a plane in a way such that a surface is entirely covered without gaps or overlaps, a 2D pattern was formed. The process was highly solvent- and concentration-dependent, because this process was driven by π–π interactions between macrocycles competing with π–solvent interactions.

**Figure 35:**
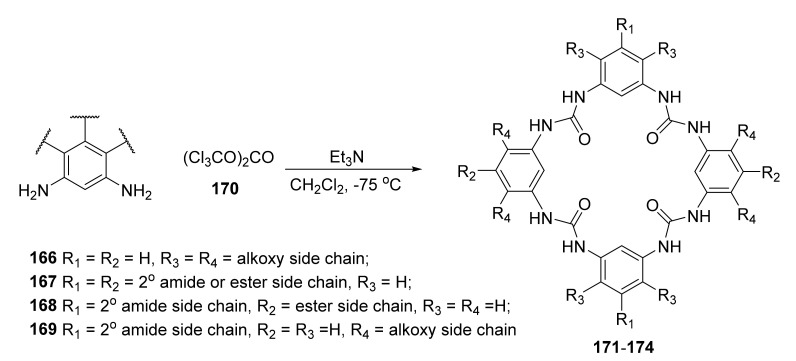
General synthetic scheme of tetraurea, *m*-phenylenediamine-containing macrocycles **171**–**174**.

Synthesis of macrocycles by the use of irreversible reaction often results in poor yields, and in many cases requires multistep synthesis. The one-pot efficient formation of the aromatic tetraurea macrocycles **171**–**174** could be conducted by the reaction of the *m*-phenylenediamine derivatives **166**–**169** with triphosgene in the presence of triethylamine (Figure 35) [59].

The factors that were crucial for efficient synthesis included a molar ratio of 2:1 of diamine to triphosgene reactants, DCM as a solvent, and temperature of −75 °C, affording yields up to 79%. Modification of this approach also allowed the researchers to obtain a tetraurea macrocycle from two different diamine components. Tetraurea macrocycles with different side chains exhibited selective cation bonding (selective toward K^+^ over Na^+^) and self-assembly behavior.

The synthesis and properties of macrocycles possessing inorganic atoms in their core structure is still a demanding task. Cyclodiphosphazane-containing macrocycles were recently explored as a candidates for supramolecular-structures building blocks, but the synthesis of larger macrocycles is still under development [60]. The traditional one-step approach usually leads to smaller macrocycles, and in the cases of asymmetric linkers, it results in mixed *exo*/*endo* arrangement in the macrocycle backbone.

**Figure 36:**
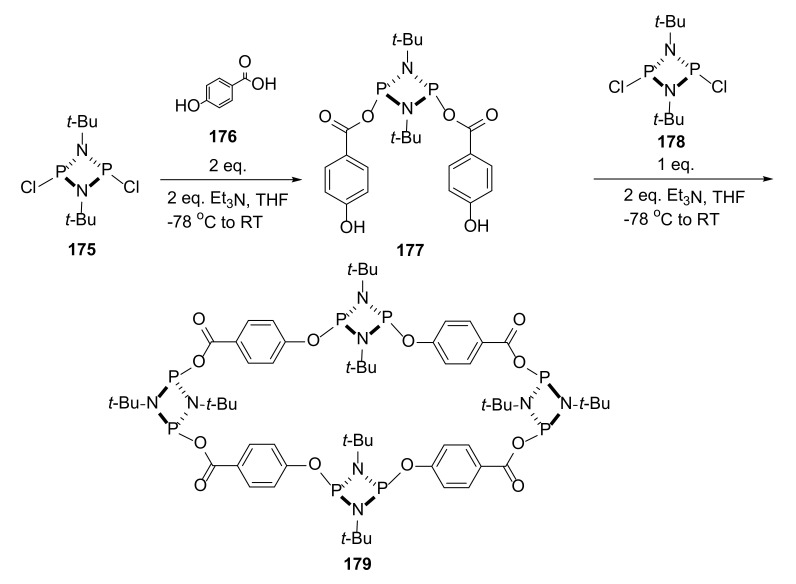
New, multistep approach to the synthesis of macrocycle **179** with prearranged building blocks.

To avoid small macrocycle formation, usually obtained in the conventional one-step approach, García and coworkers decided to use a multistep synthesis with the formation of compound **177** in the first stage of the synthesis (Figure 36). In this step, only the carboxyl group of the linker reacted, leaving free phenyl groups for the reaction in further steps. Such selectivity was achieved without the use of blocking groups, which would extend the entire synthesis. Then obtained linker was reacted with another **177** molecule, which resulted in the formation of the [4 + 4] macrocycle **179** with linkers of a specific, nonrandom, all-*cis* orientation, without the formation of a smaller [2 + 2] macrocycle. Conclusions obtained on the basis of observations of compounds formed during the synthesis were additionally confirmed by molecular modeling, resulting in a relative Gibbs free energy diagram for the formation of macrocycle **179**.

### 3.8. Other Properties

**Figure 37:**
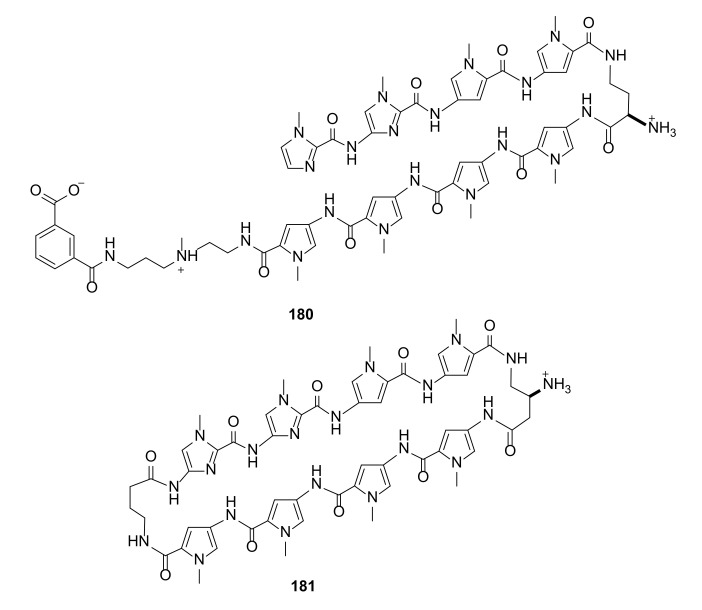
Structures of oligomeric (**180**) and macrocyclic (**181**) pyrrole–imidazole polyamides of a similar size studied in mice.

The differences in macrocycle compounds forming acyclic oligomeric analogues was the subject of application research with DNA in living organisms. The pharmacokinetic and toxic properties of macrocyclic and oligomeric pyrrole–imidazole polyamides (Figure 37) of a similar size were studied in mice [61].

Despite possessing an identical eight-ring structure, the chemical modifications were significant enough to raise different biochemical properties. The acyclic derivative **180** had a rapid bloodstream accumulation and excretion profile, whereas cyclic polyamide **181** could circulate in animals for at least two days. In addition, the tolerance of **180** and **181** differed, as the compound **180** was well tolerated by animals, while injection of **180** was lethal to the mice.

The simplest trianglimine formed from *trans*-diaminocyclohexane and terephyalic acid, synthesized more than two decades ago [62], found new applications that can be used both in the laboratory and at an industrial scale. This trianglimine was used for selective absorption of ethyl acetate in the solid state by forming solvate [63]. Formation of a stable complex was confirmed by crystal structure prediction calculations. This phenomena was observed by time-dependent solid-vapor sorption of AcOEt/EtOH 1:1 mixtures at 25 °C. Due to the size and shape of voids in its structure in a solid phase, it absorbed ethyl acetate from ethyl acetate ethanol mixtures, which are difficult to separate by normal distillation.

Crystallization of chiral tiranglimine macrocycle using a heterochiral paring strategy may introduce porosity into the crystal framework. This was observed in the macrocyclic polyiminines that do not exhibit high porosity in the enantiopure form [64]. Cooper at al proved this using the example of a simple triangilimine derived from condensation of *trans*-1,2-diaminocyclohexane and isophthalic aldehyde. Crystals of enantiopure macrocycle obtained from methanol were a solvent of molecules in the crystal lattice. After evacuation of the solvent, the polycrystalline sample exhibited no porous properties, both to N_2_ and H_2_. The crystal derived from crystallization of equimolar amounts of two enantiomeric macrocycles from ethyl acetate was stable after removal of the solvent and displayed an interconnected pore network, with the highest reported surface area for the trianglimine macrocycle SA_BET_ = 355 m^2^ g^−1^.

**Figure 38:**
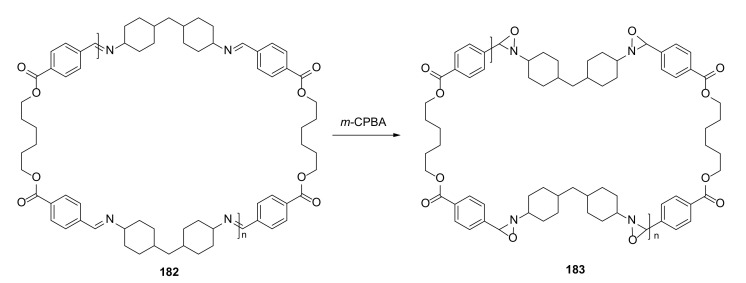
Oxidation of macrocyclic polymeric imine **182** (*n* = 1) with *m*-chloroperoxybenzoic acid.

Macrocyclic polymeric imines that could be readily obtained by polycondensation reaction were transformed into the corresponding polyoxaziridines by oxidation with *m*-chloroperoxybenzoic acid (Figure 38) [65].

The structures of the new polyoxaziridines (e.g., **183**) of different ring sizes, along with oligomeric analogues, were confirmed by NMR, MALDI-TOF MS, and IR experiments displaying conversion of total imine to oxaziridine. The synthesized polyoxaziridines were relatively stable, and could be stored at room temperature.

The interactions between macrocycles can significantly change their properties such as emission spectra and photoluminescence. These changes could be observed on the example of anthracene- and pyridinium-containing mechanically interlocked macrocycles [66]. The synthesis of this type of compound is not easy due to the Coulombic repulsion of positively charged pyridinium salts being fragments of substrate structures, but it was proven to be possible. With the emission around 675 nm in aqueous solutions, this compound may be a water-soluble cationic fluorophore with a potential application in bioimaging.

### 3.9. Catalysis

**Figure 39:**
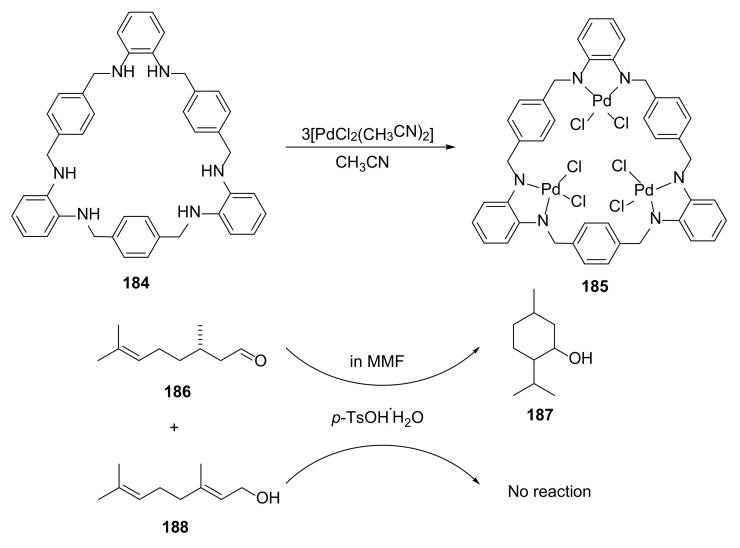
Synthesis of macrocycle **185** for the metal–macrocycle framework to catalyze specific heterogeneous cyclization reaction.

A porous metal–macrocycle framework was proved to adsorb terpenoids in a site-selective way. Crystal structures of **185** with (*S*)-citronellal, nerol, geraniol, and farnesol in a macrocyclic polyamine framework displayed different ways of binding [67]. These findings were the basis of explaining different reactivities in the cyclization reaction of terpenoids due to their arrangements in metal–macrocyclic network cavities (Figure 39).

Additionally, through several competition experiments, a substrate-specific cyclization reaction of (*S*)-citronellal in terpenoid mixtures was conducted, and displayed a strong inhibitory effect of nerol on the (*S*)-citronellal cyclization reaction.

**Figure 40:**
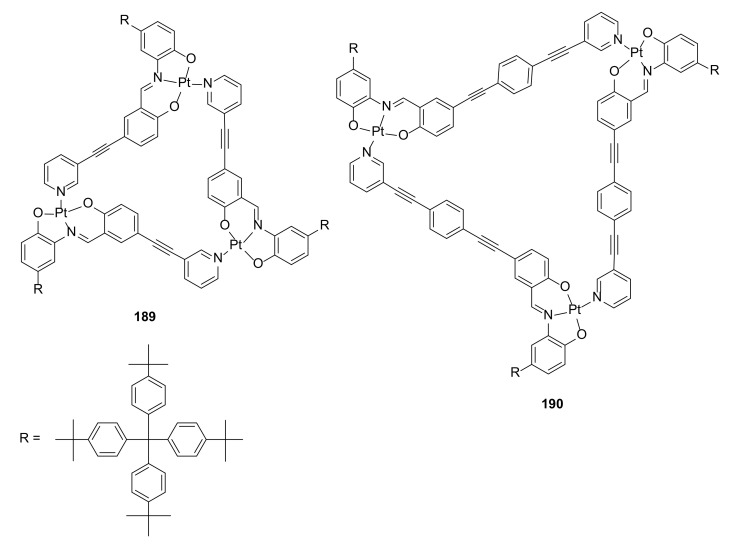
Structures of Pt_3_ macrocycles **189** and **189** obtained in Pt-bridged macrocyclization.

Nitrogen and oxygen atoms could also serve as electron-donor centers for the construction of platinum-containing macrocycles that could be used as stable catalysts. A new salicylaldehyde moiety containing a ligand with an extended conjugation multiple-bond system was synthesized, and a [3 + 3] macrocycle was formed by a reaction with K_2_PtCl_4_ (Figure 40) [68].

The macrocycle tended to aggregate at room temperature in solution and in the solid state. MALDI-TOF and TEM studies showed that these macrocycles assembled into nanotubular column structures. The incorporation of an acetylene moiety into the macrocyclic structure not only expanded the size of the linker, but also directed macrocyclization into [3 + 3] products instead of the expected [4 + 4] products observed for analogue entropy-driven reactions with shorter linkers.

## 4. Conclusions

The rapid development of chemistry of different types of nitrogen-containing macrocycles is nowadays driven by their applications, which often differs from their acyclic or oligomeric analogues. New synthetic approaches are followed by studies on their structure and potential function. This is all the more important because, as mentioned in many reports, a significant part of the syntheses of the presented macrocycles can be scaled up, which gives them industrial significance. Various structural fragments of the presented macrocyclic compounds give them interesting properties related to their behavior in solutions and in the crystal. Nitrogen atoms contained therein may serve to selectively absorb anions, organic compounds, or solvent molecules, and they may themselves serve as organocatalysts or donor atoms in metal complexes that are catalysts. The cyclic structure gives them greater rigidity, which results in greater selectivity and, in the case of optically active compounds, also stereoselectivity. Macrocyclic compounds form porous materials in which molecules of solvents or gases can be placed, and also are often selectively promising materials in organo-electronics.
